# Metallodrugs: an approach against invasion and metastasis in cancer treatment

**DOI:** 10.1002/2211-5463.13381

**Published:** 2022-04-05

**Authors:** Mauricio M. González‐Ballesteros, Carmen Mejía, Lena Ruiz‐Azuara

**Affiliations:** ^1^ Departamento de Química Inorgánica y Nuclear Facultad de Química Universidad Nacional Autónoma de México Ciudad de México Mexico; ^2^ Laboratorio de Biología Celular Facultad de Ciencias Naturales Universidad Autónoma de Querétaro México

**Keywords:** cancer, invasion, metallodrugs, metastasis, transition metals

## Abstract

Cancer is a heterogeneous and multifactorial disease that causes high mortality throughout the world; therefore, finding the most effective therapies is a major research challenge. Currently, most anticancer drugs present a limited number of well‐established targets, such as cell proliferation or death; however, it is important to consider that the worse progression of cancer toward pathological stages implies invasion and metastasis processes. Medicinal Inorganic Chemistry (MIC) is a young area that deals with the design, synthesis, characterization, preclinical evaluation, and mechanism of action of new inorganic compounds, called metallodrugs. The properties of metallic ions allow enriching of strategies for the design of new drugs, enabling the adjustment of physicochemical and stereochemical properties. Metallodrugs can adopt geometries, such as tetrahedral, octahedral, square planar, and square planar pyramid, which adjusts their arrangement and facilitates binding with a wide variety of targets. The redox properties of some metal ions can be modulated by the presence of the bound ligands to adjust their interaction, thereby opening a range of mechanisms of action. In this regard, the mechanisms of action that trigger the biological activity of metallodrugs have been generally identified by: (a) coordination of the metal to biomolecules (for instance, cisplatin binds to the N7 in DNA guanine, as Pt‐N via coordination of the inhibition of enzymes); (b) redox‐active; and (c) ROS production. For this reason, a series of metallodrugs can interact with several specific targets in the anti‐invasive processes of cancer and can prevent metastasis. The structural base of several metal compounds shows great anticancer potential by inhibiting the signaling pathways related to cancer progression. In this minireview, we present the advances in the field of antimetastatic effects of metallodrugs.

## Graphical abstract

In this review, we examine the use of metallodrugs for cancer treatment, focussing on the advances in this type of therapy and the importance of these compounds, with emphasis on Pt, Ru, Au, Zn, and Cu as antimetastatic agents. In addition, we discuss the different mechanisms of action by which metallodrugs exert their therapeutic effects.

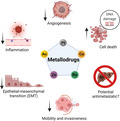

**Abbreviations**
ADAMs: a disintegrin and metalloproteinases
ATF3: activating transcription factor 3
Cas: casiopeínas®
CCN1: CCN1 family
COX‐1: cyclooxygenase‐1
CYR61: cysteine‐rich angiogenic inducer 61
DNA: deoxyribonucleic acid
EGF: epidermal growth factor
EGFR: epidermal growth factor receptor
EMT: epithelial‐mesenchymal transition
ER: endoplasmic reticulum
FN1: fibronectin 1
HPMC‐NP: nanoplatform formed of hyaluronic acid‐paclitaxel and marimastat/β‐casein complexes
IL(s): interleukins
MAP: mitogen‐activated protein
MAPK: mitogen‐activated protein kinases
MIC: medicinal inorganic chemistry
MMPs: matrix metalloproteinases
MT1‐MMP: membrane type I‐matrix metalloproteinase
NSAIDs: nonsteroidal anti‐inflammatory drugs
PGE2: prostaglandins
ROS: reactive oxygen species
SMAD3: SMAD Family Member 3
SOD: superoxide dismutase
TGF‐β: transforming growth factor‐β
TNF‐α: tumor necrosis factor α
VEGF: vascular endothelial growth factor
VEGFR‐2: vascular endothelial growth factor receptor 2
WHO: World Health Organization

## Cancer

Cancer is a group of diseases in which the constant proliferation of neoplastic cells results in tumor formation. These tumors can invade the surrounding tissues and spread to distant organs [1, 2]. There are more than 200 different types of cancer and according to the World Health Organization (WHO), cancer is a significant cause of morbidity and mortality worldwide, regardless of the level of human development. By the end of 2020, there were 19,3 million new cancer cases reported and around 10,0 million cancer‐related deaths [3]. In addition, as is mentioned by Hanahan and Weinberg in ‘The Hallmarks of Cancer’ and ‘Hallmarks of: The Next Generation of Cancer’, it is important, to recall that each cancer type has proven to be a complex, heterogeneous, and multifactorial disease, and knowledge of this provides guidelines for a better molecular understanding [2, 4], guiding the development of effective new cancer therapies. Most of the drugs against cancer are directed against a limited number of well‐established targets (proliferation or cellular death), which reflects the difficulty, time, and cost involved in identifying and validating new therapeutic targets [5]. Additionally, it is important to consider that cancer progression toward aggressive pathological stages or worse prognosis involves invasion and metastasis processes.

## Invasion and metastasis

Invasion is defined as the movement of a cell to a site or tissue normally occupied by another cell type, generally crossing a basement membrane, whereas metastasis is the establishment of a tumor cell in an organ other than the one of origin, which is not physically connected [2, 6]. Both events allow neoplastic cells to disperse, complicating treatment and causing 90% of cancer deaths [7, 8, 9, 10]. During the dissemination process, neoplastic cells follow a stepwise process known as the metastatic cascade, consisting in local invasion of surrounding tissues, intravasation, survival in the circulatory system, arrest in distant organs, extravasation, and metastasis. Neoplastic cells undergo epithelial–mesenchymal transition (EMT) in order to invade and metastasize. The transforming growth factor‐β (TGF‐β) signaling pathway is crucial, particularly during intermediate stages, to induce EMT. The TGF‐β‐induced EMT has been associated with tumor metastasis, disease recurrence, and an increase in drug and radiation resistance [11, 12]. At a cellular level, EMT is characterized by the loss of cellular adhesion and increased cellular mobility and invasiveness, which results from the cadherin switch from E‐cadherin to N‐cadherin. This switch is responsible for epithelial to fibroblast phenotypic changes, apoptosis resistance, morphological changes, cytoskeleton reorganization, and extracellular matrix degradation [10, 13, 14]. The degradation of the extracellular matrix and basement membrane is essential for invasion and metastasis development. However, some extracellular matrix components, such as interstitial collagen, are resistant to proteolytic cleavage and are only susceptible to matrix metalloproteinases (MMPs) [9, 15], such as gelatinase‐A (MMP2), gelatinase‐B (MMP9), and collagenase‐3 (MMP13). These MMPs are considered indicators of tumor aggressiveness and an unfavorable cancer prognosis [10, 16].

**Fig. 1:**
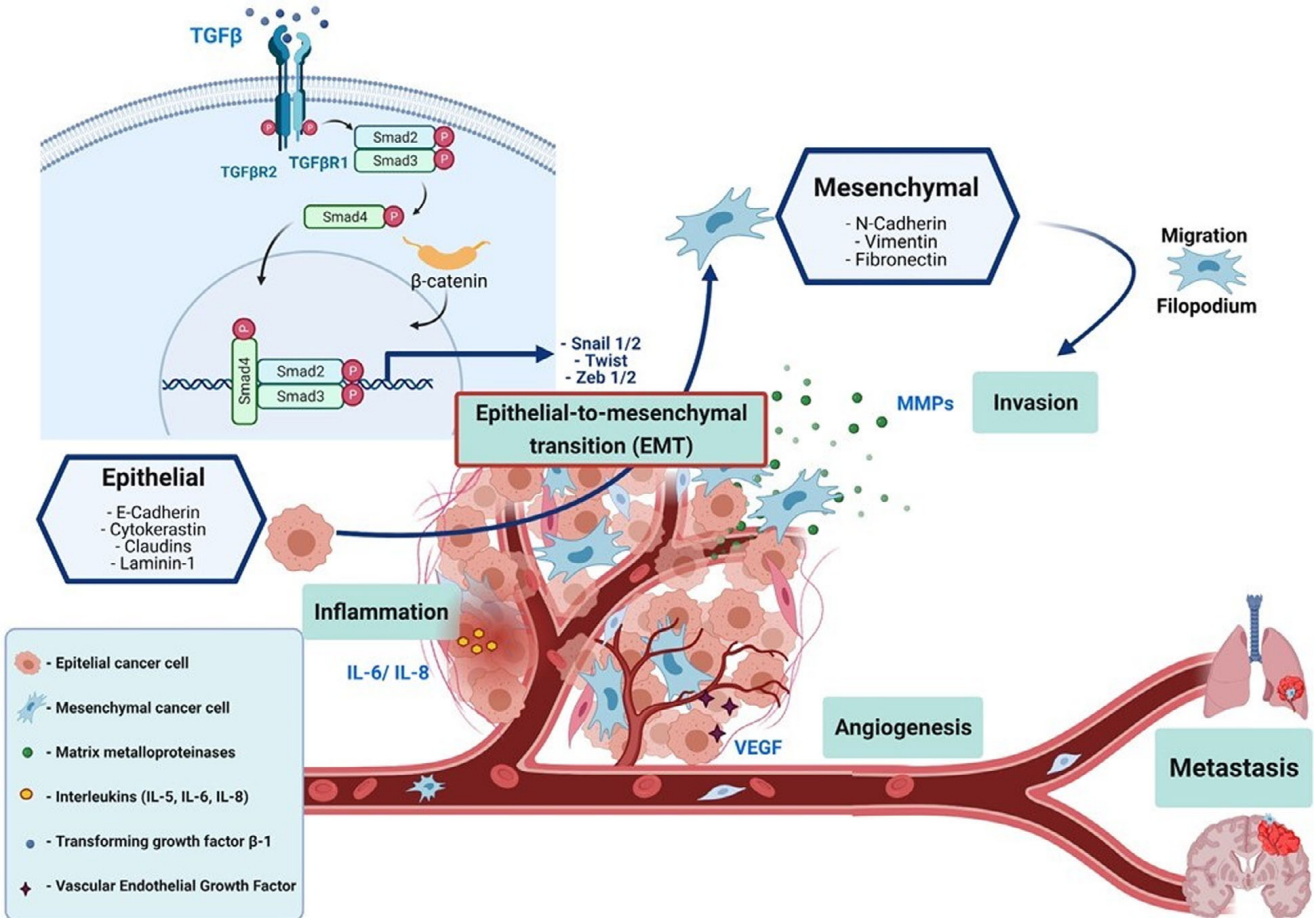
General process of metastasis. During the metastasis process, tumor cells follow a series of steps called the metastatic cascade, which consist of local invasion of nearby tissues, intravasation, survival in the circulation, arrest in distant organs, extravasation, and the establishment of metastasis. In order to carry out these processes, malignant cells lose their epithelial characteristics and acquire mesenchymal properties, this process is known as epithelial‐mesenchymal transition (EMT). At the cellular level, EMT is characterized by the loss of cell adhesion, increased mobility and invasiveness, and the secretion of extracellular matrix metalloproteases (MMPs); these changes are caused by the activation of signaling pathways such as TGF‐β or WNT / β‐catenin, increasing the expression of genes such as Snail1, Snail2, Twist, and Zeb‐1. Another key aspect for tumor development and metastasis is angiogenesis, a complex process that involves a highly regulated interaction of multiple signaling molecules. One of the most relevant pro‐angiogenic signaling molecules is vascular endothelial growth factor (VEGF) and its cognate receptor 2 (VEGFR‐2), because it promotes the formation of new blood vessels through cell migration, proliferation, and mobilization of endothelial progenitor cells; in addition, some interleukins play an important role in inflammation and the progression of metastasis, such as IL‐5, IL‐6, IL‐8, IL‐12, and IL‐17A. (Created with BioRender.com.).

Nowadays, the role of the tumor microenvironment in cancer progression is gaining significant attention. This complex interaction between the stroma and cancer cells results in a dynamic feedback loop with biochemical and biophysical signals that assist the metastatic transition of cancerous cells [12, 17]. In that context a noteworthy protein is cysteine‐rich angiogenic inducer 61 (CYR61) a member of the CCN1 family (CCN1), a matricellular protein that in humans is encoded by the *CYR61* gene. CCN1 is an important extracellular matrix protein that participates in various tumorigenic processes, such as cellular adhesion, migration, apoptosis, and angiogenesis. CCN1 regulates the endothelial overexpression of N‐cadherin by activating the nuclear translocation and signaling of β‐catenin. In turn, N‐cadherin facilitates the interaction between cancer and endothelial cells and promotes infiltration of aggressive cells into the lymphatic and blood circulation for metastasis [17]. Another key component of tumor development and metastasis is angiogenesis, a complex process involving the highly regulated interaction between multiple signaling molecules. The vascular endothelial growth factor (VEGF) and its receptor 2 (VEGFR‐2) are important pro‐angiogenic signaling molecules that exert an important role in angiogenesis and metastasis. These molecules promote neovascularization through migration, proliferation, and mobilization of endothelial progenitor cells [18]. In Fig. 1, we summarize the general process of the mechanism of metastasis.

## Metastasis as a therapeutic target

The development of new therapeutic approaches specifically designed to prevent or treat metastasis must consider the genetic and phenotypic differences of metastatic cells. An alternative approach could be to target the neoplastic cell metastatic‐enabling mechanisms, that is, protease liberation, EMT, and angiogenesis.

An example of this approach is the nanoplatform formed of hyaluronic acid‐paclitaxel and marimastat/β‐casein complexes (HPMC‐NP). HPMC‐NP reduced the expression and activity of MMPs in 4T1 and tumor cells; it also inhibited tumor growth and suppressed the development of metastasis and angiogenesis in the 4T1 metastatic breast cancer model [19].

Furthermore, the TGF‐β signaling pathway is a potential therapeutical target due to its pleiotropic functions, which regulate cellular growth, differentiation, apoptosis, motility, invasion, and angiogenesis. Inhibiting this signaling pathway could improve survival in metastatic cancer patients; Galunisertib, one of the most promising drugs in clinical phase, is a small‐molecule selective inhibitor of the TGF‐β receptor type I (RI), a serine/threonine kinase, and thus inhibits the TGF‐β signaling pathway; this drug shows potent anti‐invasive activity [20]. Additionally, Galunisertib showed antiproliferative activity in *ex vivo* models, which suggests that inhibition of TGF‐β has antitumor effects, and that the tumor microenvironment mediates these effects [20, 21]. In the same way, angiogenesis has emerged as a therapeutic target due to the importance of blood vessels in cancer development and metastasis. To date, several of the drugs used to treat different types of cancer, such as Apatinib, Axitinib, Bevacizumab, Imatinib, and Ramucirumab, inhibit angiogenesis through VEGF and other angiogenic factors [18].

Overall, metastasis represents a complex challenge due to its systemic process. The use of more than one therapeutic agent is required for its effective inhibition. Therefore, combination or multiple‐target therapies are essential to stop metastasis development.

## History of metallodrugs and their use in cancer

The use of metals for medicinal purposes has been practiced since the time of ancient civilizations, including Egyptian, Chinese, Greek, and Roman. Copper was used to sterilize wounds [22], gold to treat the skin of people affected by smallpox and skin ulcers [23], and silver to cure wounds and infections [24]. Nowadays, it is possible to find several commercial inorganic compounds for the treatment of various health problems. For example, aluminum hydroxide, aluminum carbonate, calcium carbonate, and sodium bicarbonate are used to treat stomach afflictions [25]; auranofin is prescribed to treat arthritis [26]; and bismuth subsalicylate used to treat gastrointestinal diseases [27].

Regarding cancer, treatment has three fundamental pillars: surgery, radiotherapy, and chemotherapy. The latter is the most common option, and could be a key approach to stop the metastatic process due to its systemic approach [6]. Cisplatin, an antineoplastic chemotherapy agent, is a ‘metalating’ compound with a square planar structure and a central platinum ion. The term ‘methylating’ is used for organic molecules and denotes the addition of a methyl group on a substrate, or the substitution of an atom (or group) by a methyl group; however metalating in the case of metallodrugs is the union formed between a metal atom and biomolecules to form M‐biomolecules [28, 29]. Cisplatin was the first member of the platinum‐based metallodrug family. This family also includes carboplatin and oxaliplatin, used to treat testicular, ovarian, breast, cervical, gastric, colon, prostate, and small‐cell and non‐small‐cell lung cancer [30]. Arsenic trioxide, another metallodrug, is used to treat adult patients with acute promyelocytic leukemia [31]. For example, the superoxide dismutase (SOD) biomimetic activity of the manganese‐based M‐404003 and Ca_4_[Mn(DPDP)_5_] compounds, have been evaluated in clinical studies to determine their potential use as analgesics in postsurgery treatments [32]. Other examples of clinically relevant metallodrugs are photoactivatable metal anticancer drugs and drug candidates as mentioned by Anthony et al., in 2020, for example, Photosens® (Al III), TOOKAD® soluble (Pd II), approved in clinical phase III and TLD‐1433 (Ru II), which is in clinical phase II [33].

### Platinum compounds

Throughout history, some positive health effects of inorganic compounds have been discovered serendipitously; one of the most referenced examples among inorganic chemists is the compound cis‐[PtCl_2_(NH_3_)_2_], known as cisplatin. With the initial aim of studying the influence of electric fields on the mitosis of *Escherichia coli*, Barnett Rosenberg et al., in 1960 [30], observed that bacteria stopped replicating in the presence of platinum electrodes and wrongly proposed that one of the compounds generated from the experimental materials and conditions, [(NH_4_)_2_][PtCl_6_], was responsible for the observed inhibition [34, 35]. Many experiments later they found that UV light caused a series of chemical reactions in the solution and then identified the [PtCl_4_(NH_3_)_2_] complex as the active chemical. Subsequently, knowing that neutral platinum compounds with a cis conformation are more active complexes, they altered the oxidation state of platinum to synthesize the cis‐[PtCl_2_(NH_3_)_2_] compound; this showed excellent biological activity, thus raising the prospect of studying the antitumor properties of this compound [36, 37].

**Fig. 2:**
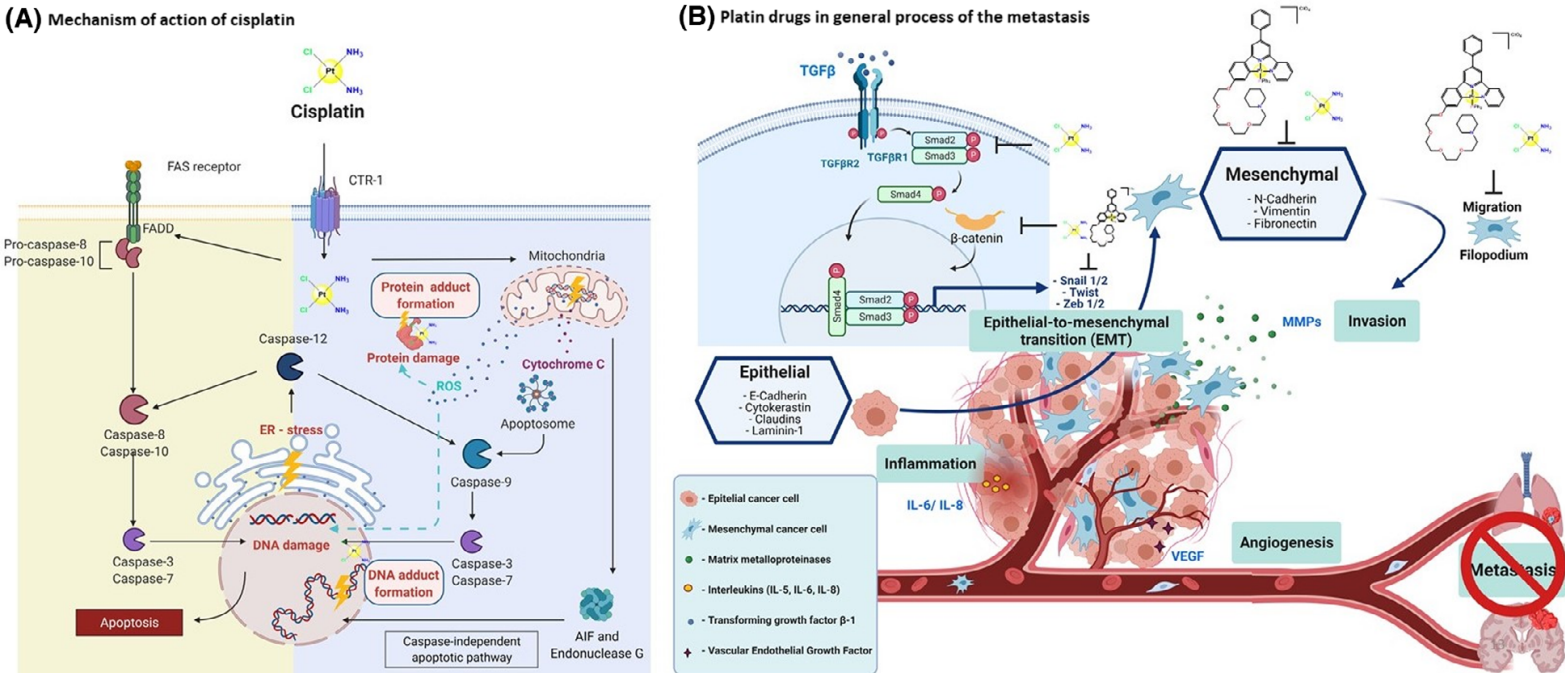
(A) Mechanism of action of cisplatin. Modern studies have revealed that copper transporter protein CTR1 is responsible for cisplatin uptake. Cisplatin activates both the intrinsic mitochondrial pathway and the extrinsic death receptor pathway of apoptosis. In addition, ER stress may also be induced. Among the three pathways, the intrinsic pathway, involving the mitochondria, is the major one. The administration of cisplatin causes cellular stress and results in the alteration of the mitochondrial membrane, leading to the release of apoptogenic factors such as apoptosis‐inducing factor (AIF), endonuclease G and cytochrome C, from the mitochondria into the cytosol. After being released from the mitochondria, endonuclease G and AIF accumulate in the nucleus, leading to apoptosis in a caspase‐independent manner while the released cytochrome C binds to the adaptor protein Apaf‐1 and induces its conformational changes, activating caspase 9, which, in turn, leads to activation of several downstream caspases for caspase‐dependent apoptosis. The ER‐stress pathway is also involved in apoptosis during cisplatin administration. Caspase 12, which localizes at the cytosolic face of the ER and is activated by ER stress, is the key initiator caspase in the ER pathway and in the extrinsic pathway. Binding of the death receptors by ligands results in the recruitment and activation of caspase 8, which leads to the activation of downstream caspases to trigger apoptosis. Furthermore, cisplatin induces oxidative stress by triggering the formation of reactive oxygen species (ROS), such as hydroxyl radical and, superoxides, which depends on the concentration of cisplatin and time of exposure. ROS are thought to be responsible for peroxidation of lipids, depletion of sulfhydryl groups, and alterations in various signal transduction pathways, which can cause DNA damage and consequently apoptosis of cells. Although cisplatin can bind to various biomolecules, it is generally considered that DNA is the major biological target, forming adducts. Adapted from ‘Apoptosis Extrinsic and Intrinsic Pathways’, by BioRender.com (2020) (Retrieved from https://app.biorender.com/biorender‐templates). (B) Platin drugs in the general process of metastasis. Cisplatin induces the expression of the transcription regulation factor ATF3, which suppresses a variety of genes related to the rearrangement of the cytoskeleton, the extracellular matrix, filopodia, and cell adhesion, including TGFβ/SMAD3 signaling and the β‐catenin signaling pathway (and consequently compromises MET), and cell migration *in vitro* and cancer metastasis *in vivo*. Similarly, PIP platinum can increase cell adhesion and block cell migration/invasion by inhibiting the Wnt signaling pathway due to nuclear translocation of β‐catenin (which is necessary for the activation of Wnt signaling) and inducing the translocation of β‐catenin in the cell membrane, which favors cell adhesion through E‐Cadherin. (Created with BioRender.com.).

Further studies focused on evaluating the effect of this compound on tumor cell lines. Eventually, the first inorganic antineoplastic compound for clinical use emerged [30]. Cisplatin activates both the intrinsic mitochondrial pathway and the extrinsic death receptor pathway of apoptosis (Fig. 2A). Cisplatin inspired numerous inorganic chemists to generate new metal‐based compounds. In the last few decades, the studies performed by inorganic chemists have allowed the development of new metal‐based drugs, thereby completing the basic and nonclinical research studies required to initiate human clinical studies.

Currently, cisplatin is one of the chemotherapy agents used to treat cancer *in situ*; it is also used in combined therapies against metastatic cancer, with favorable results. For example, Franciosi et al. (2011) in a prospective study evaluated a cisplatin/etoposide therapy against brain metastases derived from breast carcinoma, non‐small‐cell lung carcinoma, and melanoma in patients previously subjected to radiotherapy. This study observed that the cisplatin/etoposide combined therapy is effective in patients with brain metastasis derived from breast cancer and non‐small‐cell lung cancer [38].

Furthermore, in a small nonclinical study, tocilizumab increased the cytotoxic effects of cisplatin *in vitro* and *in vivo* in a triple‐negative breast cancer model by inhibiting the EMT and increasing apoptosis. These results indicate that tocilizumab/cisplatin combined therapy can suppress the metastatic effect of highly aggressive breast cancer cells [39].

Recently, a study reported various findings regarding the role of cisplatin in the EMT. Cisplatin induces the expression of activating transcription factor 3 (ATF3), which suppresses various genes associated with cytoskeleton rearrangement, the extracellular matrix, filopodia, and cellular adhesion, including the TGFβ/SMAD3 signaling pathway. This inhibition suppresses the transcription of Fibronectin 1 (FN1), which regulates the EMT and cellular migration. In a neoadjuvant chemotherapy setting, cisplatin and paclitaxel block cancer metastasis by inhibiting the colonization of target organs with neoplastic cells and cancer growth. Based on these findings, a model has been proposed in which cisplatin activates ATF3, inhibiting the positive reciprocal regulation loop between FN1 and TGF‐β, thus blocking the EMT, and thereby compromising cellular migration *in vitro* and metastasis *in vivo* [40] (Fig. 2B). Similarly, it is important to mention that in a study by He et al. 2018, carboplatin has been shown to promote apoptosis and inhibit HN‐3 cell migration. [41]

Despite its effectiveness against a considerable number of tumors, cisplatin has serious side effects, such as nephrotoxicity, myelotoxicity, ototoxicity (tinnitus), peripheral neuropathy, and gastrointestinal problems, which affect the patient's quality of life [42].

The search for less toxic treatments led to the development of new generation drugs. Carboplatin, a second‐generation drug, is used to treat ovarian, head and neck, breast, testicular, brain, gallbladder, cervical, and small‐cell lung cancer. Carboplatin is less ototoxic, nephrotoxic, and neurotoxic than cisplatin. However, the side effects associated with neurotoxicity significantly increase with age, which is why it is used to treat pediatric neuroblastoma. Additionally, hearing loss characteristic of cisplatin treatment is not observed in patients treated with carboplatin. Moreover, the main adverse effect that limits the maximum dose to be administered is thrombocytopenia [43, 44].

Oxaliplatin is less effective, as well as less cytotoxic, nephrotoxic, and ototoxic than cisplatin. Additionally, thrombocytopenia is less frequent than with carboplatin. Its most significant limitation is that it causes peripheral neuropathy, affecting the sense of touch. This is accompanied by numbness and tingling after exposure to the cold. This drug is mainly used to treat colon cancer, even after metastasis. Finally, it is important to emphasize that gastrointestinal effects are common when using this group of drugs [43, 44, 45].

Several chemotherapy agents inhibit cancer progression with serious side effects. Additionally, some types of tumors are resistant or refractory to cisplatin treatment. Therefore, the study of metal‐based compounds with antineoplastic effects has been promoted [42, 46]. Ideally, these compounds would avoid some of the most severe side effects of current chemotherapy while also seeking a possible cost reduction. Among these new metallodrugs, those composed of metals such as ruthenium, gold, zinc, and copper stand out. In this section, we will review some of the most promising metallodrug candidates.

### Ruthenium compounds

Some of the ruthenium‐based molecular complexes have an electrostatic affinity for DNA and can reversely bind to the double helix [47]. For this reason, tumor cell cycle arrest at different checkpoints is expected, resulting in apoptosis. Consequently, these molecular complexes are considered potential candidates for synthesizing new antineoplastic molecules [48]. The NAMI‐A, KP1019, and KP1339 complexes are being evaluated in clinical trials [49]. Although the mechanisms of action of these compounds are still under study, several research groups have reported interesting findings. For example, their mechanism of action is not only through DNA binding; these compounds can also generate reactive oxygen species (ROS) and inhibit protein kinases. Furthermore, these complexes have shown some selectivity and the ability to overcome the resistance faced by platinum‐based therapeutic agents.

NAMI‐A is selective for metastatic cancer cells and acts through the TGF‐β pathway [50]. KP1019, also known as FFC14A, induces significant upregulation of 284 genes and downregulation of 76 genes, some of which are associated with cell cycle arrest through ROS, mitogen‐activated protein (MAP) kinases [51], and chromatin assembly. KP1339 is being evaluated in solid tumors, such as non‐small‐cell lung cancer and colorectal carcinoma, and gastrointestinal neuroendocrine tumors. Although the mechanism of action is unclear, reports indicate that oxidative stress‐related molecules are involved [52]. However, the interaction of these compounds in metastatic systems has not been sufficiently studied.

### Gold compounds

In the last few decades, researchers have focused on the study of coated gold nanoparticles. Due to their size, optical properties, chemical stability, and biocompatibility, these nanoparticles are promising candidates for several biomedical applications, including cancer treatment [53]. Therefore, gold‐based compounds with antineoplastic potential have been recently synthesized.

Some interesting Au (I) phosphane antitumor compounds have been reported, such as [Au(d2pypp)_2_]Cl, [Au(PPh_3_)]Cl, [Au_2_(dppe)]Cl_2,_ and [Au_3_(dpmp)]Cl_3_ [26]; in addition, a heterometallic compound [(η‐C_5_H_5_)_2_TiMe(μ‐mba)Au(PR_3_)] has been reported [54], with its mode of action identified as mitochondrial dysfunction or autophagy [54, 55, 56, 57].

Bis‐[4,5‐dichloro‐(N‐methyl‐N’(2‐hydroxy‐2‐phenyl)ethyl‐imidazole‐2‐ylidene)gold(I)][dichloro‐gold] (AuL7) is a gold‐based compound with potential antimetastatic activity in the breast cancer metastatic cell line MDA‐MB‐231. This compound inhibits tubulin polymerization and topoisomerase II; it also increases oxidative stress and caspases 3, 7 and 9, which causes cellular arrest at the G2‐M checkpoint, resulting in apoptosis [58].

Furthermore, the synthesis of hybrid metallic structures to increase therapeutic efficacy has been explored due to their unusual properties compared to their building units, with different physicochemical properties [53]. The study of these hybrid compounds has broadened their application scope and improved their overall yield. The results of these studies suggest that incorporating two different biologically active metals in the same molecule improves antitumor activity due to the specific interactions between the metals and their different biological targets (cooperative effect) or the improved physicochemical properties of the heterometallic compound (synergism) [59].

### Zinc compounds

The study of new metallodrugs changed its focus to biological compounds formed by essential metals, due to the fact that there are biological mechanisms for their absorption and elimination, as well as a greater capacity for interaction with biochemical processes, particularly metals such as zinc and copper. For example, the active sites of most phosphatase enzymes have bivalent metallic ions and exhibit ease of ligand exchange, flexibility of the coordination environment, and physiological abundance (zinc is the second most abundant metal ion in biological systems).

Dasgupta et al. (2020) reported the cytotoxicity of zinc complexes as anticancer agents in different tumor cell lines, such as HCT116 (human colorectal carcinoma), HepG2 (human hepatocellular carcinoma), and A549 (non‐small‐cell lung carcinoma), with significant levels of DNA fragmentation [60].

### Copper compounds

Copper (II) metallodrugs have emerged as an attractive chemotype against cancer due to their ability to generate ROS and reactive nitrogen species, resulting in oxidative damage and cellular death. Some of these copper complexes inhibit topoisomerases or can bind to DNA. Furthermore, these complexes can also affect cell cycle checkpoints and death effector proteins [61]. Casiopeínas® (Cas), a family of copper(II)‐containing compounds, have a diimine (N‐N) type bidentate ligand in their coordination sphere, that is, phenanthroline or bipyridine; and the second charged ligand is of N‐O type (α‐aminoacidate) or O‐O donor (acetylacetonate or salicylaldehyde). Casiopeínas are soluble in water, methanol, and dextrose solution intravenous. The antitumor activity of these compounds has been described in different *in vitro* and *in vivo* cancer models [62, 63]. As for the mechanism of action, Casiopeínas induces apoptosis by increasing endonuclease G, DNA fragmentation, and activating caspase 3 [64]. They also increase mitochondrial ROS and release of cytochrome C [65]. Recently, other research groups have synthesized new copper(II)‐containing coordination compounds with a similar structure to Casiopeínas. These compounds have shown cytotoxic and antiproliferative activities in different cancer cell lines, such as osteosarcoma (HOS), breast cancer (MCF7, MDA‐MB‐231), melanoma (G361, 518A2), colon cancer (HCT‐116), cervical cancer (HeLa), ovarian carcinoma (A2780, SKOV‐3), and cisplatin‐resistant ovarian carcinoma (A2780R) [66, 67, 68]. Phenanthroline, doxycycline, and some flavonoids are among their main ligands. In Table 1, we present a list of the most studied metallodrugs for cancer treatment.

**Table 1:** Metallodrugs most studied for cancer treatment.

Metal	Compound Name/Structure	Cancer types and cell lines	IC_50_ range	Action mechanisms	Migration and invasion	Metastasis	Refs.
Platin	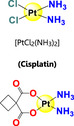 [Pt(C_6_H_6_O_4_)(NH_3_)_2_] (Carboplatin)	Breast cancer (MCF7 and MDA‐MB‐231)		DNA damage Apoptosis Generates increased ROS	Decreases fibronectin‐1 (FN1) critical for activation of the TGFβ / SMAD3 signaling pathway, Decreases the expression FN1, vimentin, and inhibits β‐catenin Inhibits migration potential and invapod formation even in cells stimulated by TGF‐β	Antitumor and antimetastatic activity in murine breast cancer model (4T1) It is conventionally used in combination with other drugs (Etoposide, Tocilizumab, etc.) to treat metastatic cancer	[39, 40, 41]
	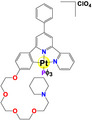 [Pt(C_35_H_40_N_3_O_4_)(C_18_H_15_P)]ClO_4_ (PIP‐platin)	Cervical carcinoma (HeLa), hepatocellular carcinoma (HepG2), breast cancer (MCF7), lung adenocarcinoma epithelial (A549), cisplatin‐resistant (A549/DDP).	8–13 µm 30.50 µm	Viability DNA damage Apoptosis Generates increased ROS	Inhibits cell migration (Scratch assay) and invasion (Transwell assay) Inhibits the WNT signaling pathway and b‐catenin translocation	Antitumor activity in murine breast cancer (4T1)	[75]
Ruthenium	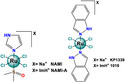 Na[RuCl_4_(C_2_H_6_OS)(C_3_H_4_N_2_)] (NAMI) (C_3_H_5_N_2_)[RuCl_4_(C_2_H_6_OS)(C_3_H_4_N_2_)] (NAMI‐A) Na[RuCl_4_(C_7_H_6_N_2_)_2_] (KP1339) (C_3_H_5_N_2_)[ RuCl_4_(C_7_H_6_N_2_)_2_] (1019)	Cervical carcinoma (HeLa) Colon (HCT116 and SW480)		Generation of reactive oxygen species (ROS) in HCT116 and SW480 cells	Transcriptome	Antitumor and antimetastatic activity in a murine model of ovarian cancer	[29, 71]
	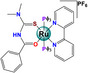 [Ru(C_18_H_15_P)_2_(C_10_H_12_N_2_OS)(C_10_H_8_N_2_)]PF_6_ (Ru(PPh_3_)_2_(O‐S)(bpy)PF_6_	Breast cancer (MCF7, MCF‐10A, and MDA‐MB‐231)	8.81‐14.82 µm	Viability Apoptosis (Caspase 3 and DNA damage)	Reduces the expression of β1‐integrin, EGFR, p38 MAPK, and the activity of MPP2		[81]
	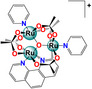 _[Ru3(O)(C2H3O2)5(C12H7N2)(C5H5N)2]PF6_ [Ru_3_(O)(OAc)_5_(phen^‐^)(py)_2_]PF_6_	Murine melanoma (B16F10)	25 µm	Interaction with the DNA double helix.			[79]
Ruthenium	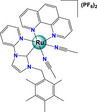 _[Ru(C20H24N3)( C12H8N2)(CH3CN)2](PF6)2_ [Ru(N‐C)(phen)(CH_3_CN)_2_](PF_6_)_2_ Ru8	Lung cancer (A549, A549 / cisR) Ovarian cancer (A2780) Liver cancer (Huh‐7) Murine melanoma (B16‐F10)	6.9‐25 µm	Viability Cell cycle arrest Apoptosis (caspase 9 and caspase 3)	Decreases expression of MPP9 and EGFR‐p Inhibits angiogenesis	Decreases angiogenesis in chicken embryos. Antitumor and antimetastatic activity in a murine model Ovarian cancer	[71]
	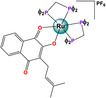 [Ru(C_15_H_13_O_3_)( C_25_H_22_P_2_)_2_]PF_6_ Ru(Lap)(dppm)_2_]PF_6_	Breast cancer MDA‐MB‐231	2.7 µm	Apoptosis Affects mitochondrial membrane potential	Inhibits cell migration and chemotaxis (Scratch assay Boyden‐Chamber Assay)		[80]
	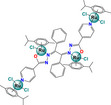 [Ru_4_(C_10_H_14_)_4_(C_24_H_18_N_4_O_2_)Cl_6_] Ru1 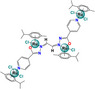 [Ru_4_(C_10_H_14_)_4_(C_14_H_10_N_4_O_2_)Cl_6_] Ru2	Cancer de pulmón (A549, A549cisR) Mama (MCF‐7) colon (LoVo) Hígado (HuH‐7)	1.39– 17.24	Apoptosis and cell cycle detection	Inhibits cell migration and invasion (Scratch assay and Transwell assay)	Less toxicity in contrast to cisplatin	[69]
Ruthenium	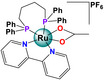 [Ru(CH_3_CO_2_)(C_28_H_28_P_2_)(C_10_H_8_N_2_)]PF_6_ [Ru(OAc)(dppb)(bipy)]PF_6_	Breast cancer (MCF7, MCF‐10A and MDA‐MB‐231)	31.6–200 µm	Affects cell morphology and cytoskeleton structure Apoptosis (Caspase 9, Caspase 3 and BCL‐2) Interaction with DNA	Inhibits cell migration, invasion, and chemotaxis (Scratch assay, Boyden‐Chamber assay and Transwell assay) Reduces MPP2 activity		[70]
Gold	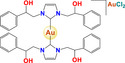 [Au(C_38_H_42_N_2_)_2_][AuCl_2_] AuL6 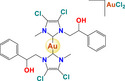 [Au(C_22_H_26_N_2_][AuCl_2_] AuL7	Breast cancer (MDA‐MB‐231)	2.10 µm	Increase in reactive oxygen species Cell cycle arrest in G2 / M Activation of caspases 3/7 and 9	Inhibits tubulin polymerization and topoisomerase II activity		[58]
	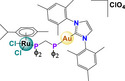 [Cl_2_(C_10_H_14_)Ru(C_25_H_22_P_2_)Au(C_21_H_24_N_2_)]ClO_4_ RANCE‐1	Human clear‐cell renal cell carcinoma (Caki‐1)	8.7 µm	Viability Apoptosis Cell cycle arrest	Inhibits cell migration and invasion (Scratch assay and Transwell assay) Inhibition of VEGF secretion		[66]
Gold	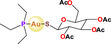 [Au(C_6_H_15_P)(C_14_H_19_O_9_S)] Auranofin 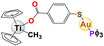 [(C_5_H_5_)_2_(CH_3_)Ti(C_7_H_4_O_2_S)Au(C_18_H_15_P)] Titanocref 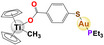 [(C_5_H_5_)_2_(CH_3_)Ti(C_7_H_4_O_2_S)Au(C_6_H_15_P)] Titanofin	Clear cell renal cell carcinoma (Caki‐1)	0.097– 2.8 µm	Viability Apoptosis Cell cycle arrest	Inhibits cell migration, chemotaxis and invasion (Scratch assay and Transwell assay) Inhibits angiogenesis: in human umbilical vein endothelial cells (HUVEC) in an ECM‐like matrix based on the length of the uninterrupted tubes (TL) and the number of branch points or nodes in the tubes (TN) Inhibition of VEGF Decreases the expression of pro‐inflammatory and prometastatic cytosines (TNF‐α, and interleukins) also decreases the expression of MMPs		[78]
Zinc	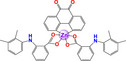 [Zn(C_12_H_6_N_2_O_2_)(C_15_H_14_NO_2_)_2_] [Zn(phendione)(MFN)_2_] 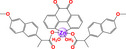 [Zn(C_12_H_6_N_2_O_2_)(C_14_H_14_O_3_)_2_(H_2_O)_2_] [Zn(phendione)(NPR)_2_(H_2_O)_2_]	Breast cancer (MDA‐MB‐231)	≈ 1 µm	Caspase‐mediated apoptosis (Caspase 3, 8, and 9)	Inhibits cell migration (Scratch assay) Decreases Vimentin and β‐Integrin Inhibits EMT Anti‐inflammatory Selective inhibition of COX‐1 and Prostaglandins		[82]
Zinc	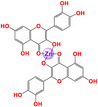 [Zn(C_15_H_9_O_7_)_2_] Q‐ZnCPX	Urinary bladder transitional cell carcinoma (BFTC‐905)	≈ 75 µm	Viability and proliferation	Inhibits cell migration, chemotaxis and invasion (Scratch assay and Transwell assay) Decreases MMP14 and AKT‐P PI3K / AKT / mTOR pathway		[83]
	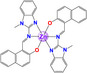 [Zn(C_19_H_14_N_3_O)_2_] [Zn(bimnap)_2_] 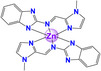 [Zn(C_12_H_10_N_5_)₂] [Zn(BnI)₂]	Liver cancer (HepG2), skin cancer (SK‐MEL‐1), colon cancer (HT018) cervical cancer (Hela) and breast cancer (MDA‐MB‐231)	6.66– 15.8 µm	Viability Apoptosis	Inhibits chemotaxis and cell invasion (Transwell assay)		[73]
			19–26.7 µm	Viability Apoptosis	Inhibits chemotaxis and cell invasion (Transwell assay)		[72]
Copper	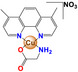 [Cu(C_2_H_4_NO_2_)(C_14_H_12_N_2_)]NO_3_ Casiopeína II‐gly	Ovarian cancer (CH1) Murine leukemia (L1210) Cervical cancer (HeLa, SiHa and CasKi) Breast cancer (MCF‐7) Colon (HCT15) Glioblastoma (C6, U87MG) Murine melanoma (B16) Lung cancer (A549, H157 and SKLU) and Neuroblastoma (CHP‐212, SK‐N‐SH)	1 µm– 10 µm	Inhibits viability and proliferation Apoptosis (Caspase‐3, Endonuclease G, Cytochrome C) Increased ROS in mitochondria	Reduces the expression of proteins such as β‐catenin, GSK‐3β, Dvl affecting the Wnt signaling pathway Decreases the expression of genes related to migration such as TGFβ‐R1, AURKA, SNAI2, BMP4, BMP6, and N‐Cadherin		[42, 87, 88, 89]
	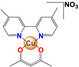 [Cu(C_5_H_7_O_2_)(C_12_H_12_N_2_)]NO_3_ Casiopeína III‐ia		10 – 60 µm		Inhibits cell migration		[86]
	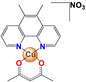 [Cu(C_5_H_7_O_2_)(C_14_H_12_N_2_)]NO_3_ Casiopeína III‐La	Cervical cancer (HeLa, SiHa) Colon (HCT15) Brain Tumor (glioma U373) Breast cancer (MCF‐7)	1.7–4.3 µm		Exerts an antiproliferative effect, promoting apoptotic cell death and inactivating the invasive process (wound healing and Transwell invasion assays) by generating ROS, inactivating GSK3β, activating JNK and ERK, and promoting the nuclear accumulation of β‐catenin. Decrease the levels of MMP9 and MMP2 and increases E‐cadherin expression		[63, 92]
	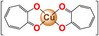 [Cu(C_7_H_5_O_2_)_2_] [Cu(trp)_2_]	Breast cancer (MCF7 and MDA‐MB‐231)	4–5.2 µm	Viability Apoptosis	Decrease MMP2 and MPP9 Inhibits cell migration and 3D cell culture invasion (Scratch assay)		[74]
	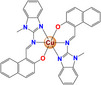 [Cu(C_12_H_10_N_5_)₂] [Cu(BnI)₂]	Liver cancer (HepG2), skin cancer (SK‐MEL‐1), colon cancer (HT018) cervical cancer (Hela) and breast cancer (MDA‐MB‐231)	3.5–17.8 µm	Viability Apoptosis	Inhibits chemotaxis and cell invasion (Transwell assay)		[72]
Copper	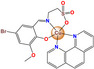 [Cu(C_10_H_10_NO_5_SBr)(C_12_H_8_N_2_)] [Cu(O‐N‐O)(phen)]	cervical cancer (Hela and C33A)	3.5–17.8 µm	Viability/ Apoptosis (BCL‐2, Bax and Caspase 3)	Inhibits cell migration, chemotaxis, and invasion (Scratch assay and Transwell assay) Inhibits cell migration and 3D cell culture invasion Reduces the expression of VEGFR‐2, FAK, AKT, AKT‐p	*In vivo* antitumor activity (mice) and antiangiogenic (chicken embryos)	[93]
	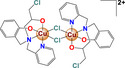 [Cu_2_(C_16_H_17_N_2_O_2_Cl)_2_Cl_2_] [Cu_2_(BPClNOL)_2_Cl_2_]	Neuroblastoma (H4)	N/A	Cell cycle arrest	Inhibits chemotaxis and cell invasion (Transwell assay) Reduces invasion in 3D cultures, reduces gene expression: snail vimentin and increases E‐cadherin expression		[85]
	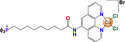 [Cu(C_39_H_39_N_3_OP)Cl_2_]Br [Cu(CPT8)Cl_2_]Br	Cervical cancer (HeLa), ovarian cancer (SKOV‐3), kidney cancer (HK‐2), and melanoma (B16F10)	6.5– 21.57 µm	Viability/Cell cycle arrest	3D cell culture invasion Reduces the expression of MMP2 and VEGFR1		[67]
Copper	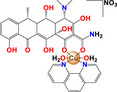 [Cu(C_22_H_22_N_2_O_8_)(C_12_H_8_N_2_)(H_2_O)_2_]NO_3_ [Cu(doxycycline)(phen)(H_2_O)_2_]NO_3_	Cervical cancer (HeLa) and breast cancer (MCF‐7)	0.5– 0.87 µm	Viability	Inhibits cell migration (Transwell assay) Reduces the expression of MMP2		[66]
	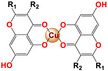 [Cu(R_1_R_2_C_9_H_3_O_4_)_2_]	Melanoma (518A2), colon cancer (HCT‐116), cervical cancer (KB‐V1 / Vbl), breast cancer (MCF‐7) and skin fibroblasts (HF).	6.7– 50 µm	Viability/Cell cycle arrest	Inhibits cell migration (Scratch assay) Reduces the expression of MMP2 and MMP7 β‐actin		[68]

## Metallodrugs against migration and metastatic processes

Several studies have reported the inhibition of cancer migration and metastasis processes using metallodrugs. For example, ruthenium complexes and arene ligands have been successfully applied in different cancer cell lines, such as lung carcinoma (A549), MCF‐7, colon adenocarcinoma (LoVo), and hepatocarcinoma (HuH‐7), decreasing viability and proliferation, and inducing apoptosis. Furthermore, these complexes also decrease cellular migration and invasion [69]. Another ruthenium‐based compound, [Ru(OAc)(dppb)(bipy)]PF_6_, induces apoptosis (through activation of caspases −9 and −3, DNA interaction, and Bcl‐2 subexpression) and affects cellular morphology and cytoskeleton structure; it also decreases migration, chemotaxis, invasion, and MPP2 activity [70]. Additionally, Wang et al. (2020) reported that compound Ru8 efficiently inhibits the metastatic process against tumor cell invasion and migration, but also has potent antiangiogenic effects *in vitro* and *in vivo* models. In a metastatic A2780 tumor xenograft‐bearing mouse model, Ru8 administration outperformed NAMI‐A compound and cisplatin in its antitumor potential and metastasis inhibition capacity (Table 1) [71].

Turning to zinc complexes, one with ‘bimnap’ type ligands derived from 1‐methyl‐2‐aminobenzimidazole and 2‐hydroxynaphthaldehyde, and another with a new ligand called BnI derived from benzimidazole and the zinc(II)complex, [Zn(BnI)₂], [72] have anticancer activity in five different human tumor cell lines: HepG2 (liver), SK‐MEL‐1 (skin), HT018 (colon), HeLa, and MDA‐MB‐231, decreasing viability and inducing apoptosis; these compounds also decrease chemotaxis‐induced migration [72, 73].

Among the copper‐containing complexes, [Cu(trp)_2_] decreases the viability of MCF‐7 and MDA‐MB‐231, induces apoptosis and decreases migration and invasion of 3D cell cultures, and decreases the formation of breast spheres and the expression of MMP2 and MPP9 [74]. Similarly, other copper‐containing metallodrugs also decrease the expression of these metalloproteinases and MMP9. The main ligands of these compounds are phenanthroline, doxycycline, and flavonoids. Some of these compounds exerts anti‐invasion activity in models *in vitro* [66, 67, 68]. [Cu(BnI)₂], [72] which has the same Bnl ligands, also has anticancer activity in different cancer cell lines, inducing apoptosis and decreasing migration capacity.

The evidence is very diverse and shows that metallodrugs can decrease the ability of tumor cells to migrate or invade. However, the mechanisms of action and cellular effects of these compounds require further analysis. Some metallodrugs have been studied in greater depth, increasing understanding of their participation against invasion and metastasis processes.

### Proposed mechanisms of action

It is essential to continue studying novel platinum complexes to overcome drug resistance and improve pharmacological activity. An important example is PIP‐platinum, which inhibits the proliferation of several tumor cell lines, including a cisplatin‐resistant cell line of adenocarcinomic human (A549/DDP). This complex triggers mitochondrial dysfunction, cytochrome c release, and increases ROS, thus inducing apoptosis. Additionally, PIP‐platinum increases cellular adhesion and decreases cellular migration/invasion in three *in vitro* models by inhibiting the WNT signaling pathway that blocks nuclear translocation of β‐catenin, which promotes E‐cadherin‐mediated cellular adhesion (Fig. 2B). Of note, PIP‐platinum also showed antitumor activity in a breast cancer murine model (4T1) [75].

Another strategy employed by Pt drugs, namely, immunogenic cell death induced by photoactive Pt complexes, might be important for antimetastatic activity [76].

Moreover, it is important to mention that some complexes of other metals overcome the resistance to Pt, since they have different target sites or the resulting DNA damage is different; as a result, the potential of heterometallic compounds as cancer therapy is currently being explored. [59]. Elie et al. (2019) [77] compared the activity of two bimetallic compounds and one monometallic gold‐based compound in a kidney metastatic carcinoma cell line (Caki‐1). This study reported that three evaluated compounds: auranofin, titanofin, and titanocref (gold‐ and titanocene‐based metallic compounds), induce apoptosis; titanofin and titanocref are more efficient at arresting the cell cycle at G2/M, while titanocref is a more efficient inhibitor of cellular migration. Furthermore, the three compounds can inhibit angiogenesis. Although the signaling pathways by which these compounds act are not clear, markers associated with the processes of migration, invasion, and angiogenesis were detected, such as prometastatic IL(s), MMP(s), TNF‐α, and VEGF [77].

Another hybrid compound with potential antineoplastic activity belongs to the ruthenium‐gold family. This complex incorporates heterocyclic Au‐N ligands derived from [Cl_2_(p‐cymene)Ru(μ‐dppm)Au(NHC)]ClO_4_, known as RANCE‐1. Compared to auranofin, this compound reduces cellular migration and invasion more efficiently. This effect is accompanied by the modification of the levels of proteins related to oxidative stress and metastasis; this includes reduction of the mitochondrial protein TrxR, the angiogenesis mediator factor VEGF, the secretion of metalloproteinases (MMP‐1, MMP‐3, MMP‐7, MMP‐8, MMP‐9, MMP‐10, MMP‐12, and MMP‐13), ADAM proteolytic enzymes (ADAM8 and ADAM9), and proteases from the cathepsin family (B, D, L, S, and Z/X/P). Furthermore, this complex decreases the levels of inflammation and metastasis‐related interleukins (IL‐5, IL‐6, IL‐8, IL‐12, and IL‐17A). However, its mechanism of action is still unknown [77, 78].

Regarding ruthenium‐based compounds, those with PF_6_ as a counter ion are of great interest for the development of selective antineoplastic therapies in metastatic stages. The PF_6_ compounds with promising results are [Ru_3_(μ_3_‐O)(μ‐OAc)_5_(μ‐η^1^(C),η^2^(N,N)‐phen)(py)_2_]PF_6_, and [Ru(Lapachol)(bis(diphenylphosphine)methane)_2_]PF_6_. [Ru_3_(μ_3_‐O)(μ‐OAc)_5_(μ‐η^1^(C),η^2^(N,N)‐phen)(py)_2_]PF_6_ shows selective cytotoxicity in the murine melanoma cell line B16F10 compared to the fibroblast L929 cell lines at a concentration of IC50 = 25 µm. Although the mechanism of action remains unknown, it has been shown that this compound interacts with DNA through electrostatic attraction [79]. [Ru(Lapachol)(bis(diphenylphosphine)methane)_2_]PF_6_ induces mitochondrial apoptosis at a concentration of IC50 = 0.13 µm in the metastatic breast cancer cell line MDA‐MB‐231. Although no tissue selectivity was observed when compared with the MCF‐10A breast epithelial nontumor cell line, it shows greater tolerance to the effect of the compound at an IC50 of 2.7 µm [80]. However, Becceneri et al. (2020) reported that the Ru(PPh_3_)_2_(O‐S)(bpy)PF_6_ complex is cytotoxic and selective to triple‐negative breast cancer cells in 3D cultures, because has the ability to reduce the expression of β1‐integrin, EGFR, and p38 MAPK and decrease the activity of MPP2 [81].

Recently, Deb et al. (2020) [82] reported the synthesis, characterization, and biological evaluation of a zinc(II)‐naproxen and a zinc(II)‐mefenamic acid complex of 1,10‐phenanthroline‐5,6‐dione [82]. Both complexes exhibit antiproliferative activity in the human breast cancer cell line MDA‐MB‐231. The zinc(II)–naproxen complex induces apoptosis through activation of caspases −3, −8, and −9. Additionally, the anti‐inflammatory properties of nonsteroidal anti‐inflammatory drugs (NSAIDs) are also conserved in metallic complexes since both complexes inhibit the cyclooxygenase pathway (selective inhibition of COX‐1 at low concentration), decreasing the synthesis of prostaglandins (PGE2), which shows anti‐inflammatory activity. *In vitro* cell migration and EMT‐related genes (VIMENTIN and β1‐INTEGRIN) also decrease. Mechanistic studies indicate that ternary complexes are more active than cisplatin and can overcome cisplatin resistance in MDA‐MB‐231 cells [82].

**Fig. 3:**
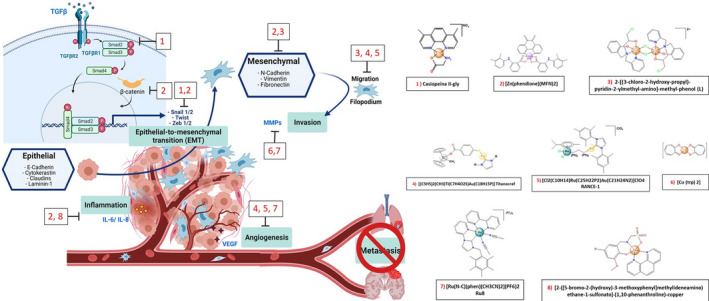
Metallodrugs in the general process of the invasion and metastasis. In order to undergo invasion and metastasis, malignant cells require different processes, which allow them to acquire invasive properties (mobility, degradation of the extracellular matrix, etc.); these changes are caused by the activation of signaling pathways such as TGF‐β or Wnt/β‐catenin, increasing the expression of genes such as Snail1, Snail2, Twist, and Zeb‐1. It also needs to mold its microenvironment by means of signaling molecules such as VEGF or EGF (angiogenesis) and interleukins that have an important role in inflammation and the progression of metastasis (such as IL‐5, IL‐6, IL‐8, IL‐12, and IL‐17A). To date, different metallodrugs have been described that can intervene or inhibit these processes. (Created with BioRender.com.).

Quercetin is a polyphenolic flavonoid with demonstrated anticancer effect. The quercetin‐zinc (Q‐ZnCPX) complex slightly reduces the viability of bladder cancer cells (BFTC‐905) at high concentrations. However, at low concentrations, Q‐ZnCPX drastically reduces cellular movement and the expression of invasion‐related proteins. The effect on cellular migration and invasion of Q‐ZnCPX was determined in three *in vitro* models. These processes were significantly decreased compared to the control, with concentrations ≥ 12,5 μM through the regulation of p‐AKT and MT1‐MMP [83]. AKT was described as an essential protein of the Akt / PI3K / PTEN signaling pathway; it is a serine/threonine protein kinase that, once activated through phosphorylation (p‐AKT), plays an important role in cancer progression [84] and metastasis. Meanwhile, MT1‐MMP (membrane type I‐matrix metalloproteinase) is one of the proteinases involved in cell migration. Among the copper metallodrugs, those containing the 2‐[(3‐chloro‐2‐hydroxy‐propyl)‐pyridin‐2‐ylmethyl‐amino]‐methyl‐phenol (L) ligand were reported to induce cell cycle arrest and decrease chemotaxis‐induced migration in neuroblastoma cells (H4). Additionally, this compound inhibits migration in 3D cultures, reduces the expression of EMT‐related genes, such as SNAIL and VIMENTIN, and increases E‐CADHERIN expression, enhancing cell adhesion [85]. Similarly, Cas III‐ia, besides showing a dose‐dependent antiproliferative activity in different tumor cell lines, can also decrease migration in the neuroblastoma cell line SK‐N‐SH [86]. Furthermore, the expression of genes induced by Cas II‐gly treatment were studied in HeLa cells, demonstrating that this compound can decrease the expression of migration‐related genes, such as: TGFβR1, AURKA, SNAI2, BMP4, BMP6, and N‐CADHERIN [42, 86, 87, 88]. Cas II‐gly was also shown to act on MALAT1, targeting miR‐17‐5p to inhibit FZD2 expression by inactivating the Wnt signaling pathway, thus inhibiting cell proliferation and promoting apoptosis in HeLa and CaSki cells [89]. This is important, since invasion and metastasis require the EMT. Particularly, the TGF‐β and Wnt signaling pathways are crucial to induce the EMT. Moreover, previous studies have reported that regardless of the mechanism of inhibition of the TGF‐β pathway and the EMT, motility, invasion capacity, and migration are decreased in different cancer cell lines [21, 90, 91]. It is important to highlight that Castillo‐Rodríguez et al. (2021) [92] reported that Casiopeína III‐La, in addition to its antiproliferative and pro‐apoptotic effects, has the ability to decrease the invasive capacity of glioma cells, through the induction of ROS and regulation of the Wnt/β‐catenin pathway [92]. Other interesting compounds include: CPT8, since it affects viability and cell cycle arrest in different cancer cell lines and reduces migration and invasion capacity in different *in vitro* models. Interestingly, this compound also decreases the expression of VEGF receptors [67, 93]. In a murine model, CTP8 showed antitumor activity and antiangiogenesis activity in a chicken embryo model [93]. In Fig. 3, we present a summary of some of the most promising metallodrugs for targeting metastasis.

## Conclusion

Since the emergence of the field of inorganic medicinal chemistry, various new drugs with a metal base or metallodrugs have been developed to combat various diseases such as cancer, specifically in those high‐risk tumors that develop invasive and metastatic properties.

In this review article, we described the mechanism of action of various platinum, ruthenium, gold, zinc, and copper‐based metal drugs. These mechanisms include effects on cell viability and apoptosis, cell cycle arrest, alteration of the cytoskeleton, inhibition of angiogenesis, and DNA damage. The current data from metallodrugs support the idea they may function as chemotherapeutic agents, because of their potential to inhibit signaling pathways for multiple aspects of cancer progression including tumor growth, angiogenesis, and metastasis.

It must be remembered that to understand how the pathways described above are altered by metallodrugs, it has been necessary to develop exhaustive analyses of cell biology to establish which cell organelles are capable of acting as biological targets. Thus, for example, we know that the permeability of the transition pore for mitochondria can be altered, which favors the generation of ROS and the entry of proteins such as Bax or the exit of Bcl‐2 and cytochrome C, which results in apoptosis. The formation of autophagosomes is also promoted to protect damaged cellular material or cause its degradation when they bind to lysosomes. Finally, in the cell nucleus, DNA is altered, as occurs with cisplatin, which fosters unions between the chains of the double helix, directly inhibiting DNA synthesis.

The use of various state‐of‐the‐art techniques, such as advanced synchrotron cryotechniques of light have made it possible to visualize the 3D cytoarchitecture in the entire cell with minimal disturbance, showing for example that iridium is 100 times more powerful than cisplatin.

Finally, metallomics allows the identification of genes that could be considered as pharmacological targets by virtue of a drug that acts on it. This has allowed the development of platforms such as drug target, which integrates information from at least 15 pharmacological databases that include information on drugs, drug targets, type of drug–target interaction, data sources, and other characteristics.

Undoubtedly, there is still a need for more research on current metallodrugs or even the development of new metal drugs.

## Conflict of interest

The authors declare no conflict of interest.

## Author contribution

MMG‐B: performed references research in data base formal analysis, investigation, writing—original draft, and visualization. CM performed formal analysis, review, and editing, LR‐A performed conceptualization, supervision, and writing—review and editing project administration.

## Data accessibility

Data accessibility as open access.
